# How proximal femur fracture patients aged 65 and older fare in survival and cause of death 5+ years after surgery: A long-term follow-up

**DOI:** 10.1097/MD.0000000000033863

**Published:** 2023-05-17

**Authors:** Kazuhiko Hashimoto, Yu Shinyashiki, Kazuhiro Ohtani, Ryosuke Kakinoki, Masao Akagi

**Affiliations:** a Department of Orthopedic Surgery, Kushimoto Municipality Hospital, Wakayama, Japan; b Department of Orthopedic Surgery, Kindai University Hospital, Osaka-Sayama City, Osaka, Japan.

**Keywords:** cause of death, older adults, proximal femur fracture, survival

## Abstract

Although the incidence of proximal femur fractures (PFFs) is increasing, few detailed reports on associated long-term outcomes and causes of death exist. We aimed to evaluate long-term outcomes and causes of death ≥5 years after surgical treatment of PFFs. This retrospective study included 123 patients (18 males, 105 females) with PFFs treated at our hospital between January 2014 and December 2016. Cases (median age: 90 [range, 65–106] years) comprised 38 femoral neck fractures (FNFs) and 85 intertrochanteric fractures (IFs). Surgical procedures included bipolar head arthroplasty (n = 35), screw fixation (n = 3), and internal fixation with nails (n = 85). The mean post-surgical follow-up time was 58.9 (range, 1–106) months. Surveyed items included survival (1 vs 5 years; sex; age, >90 vs <90 years; IF vs FNF), comorbidities, waiting time after the injury (died vs survived), operative time (proximal femoral nail antirotations [PFNA] vs FNF, died vs Survived), blood loss (PFNA vs FNF; died vs survived), and cause of death (IF vs FNF; <1 vs >1 year). Among all patients, 83.7% had comorbidities (IF, 90.5%; FNF, 81.5%). Among patients who died and survived, 89.1% and 80.5% had comorbidities, respectively. The most common comorbidities were cardiac (n = 22), renal (n = 10), brain (n = 8), and pulmonary (n = 4) diseases. Overall survival (OS) rates at 1 and 5 years were 88.9% and 66.7%, respectively. Male/female OS rates were 88.8%/88.3% and 66.6%/66.6% (*P* = .89) at 1 and 5 years, respectively. OS rates for the <90/≥90 age groups were 90.1%/76.7% and 75.3%/53.4 (*P* < .01) at 1 and 5 years, respectively. The 1- and 5-year OS (IF/FNF) rates were 85.7%/88.8% and 60%/81.5%, respectively; patients with IFs had significantly lower OS than those with FNFs at both timepoints (*P* = .015). There was a marked difference in the operative time between died (43.5 ± 24.0: mean ± S.D.) and survived (60 ± 24.4: mean ± S.D.) patients. The main causes of death were senility (n = 10), aspiration pneumonia (n = 9), bronchopneumonia (n = 6), worsening heart failure (n = 5), acute myocardial infarction (n = 4), and abdominal aortic aneurysm (n = 4). Overall, 30.4% of the cases were related to comorbidities and related causes (e.g., hypertension-related ruptured large abdominal aneurysm). Managing comorbidities may improve long-term postoperative outcomes of PFF treatment.

## 1. Introduction

In recent years, we have become a rare aging society, and the number of older individuals has continued to increase.^[1]^ As the population ages, the number of patients with proximal femur fractures (PFFs) increases.^[2,3]^ The number of PFFs is expected to increase from 1.7 million in 1990 to over 6 million by 2025.^[2,3]^ PFFs in older patients can lead to complications, such as pulmonary embolism, infection, and heart failure, with a fatality rate of approximately 10% within 1 year.^[4,5]^ Mortality^[6,7]^ and fatal complications^[8,9]^ have been reported in several studies. However, there are few reports on long-term outcomes and few detailed reports on the causes of death. Therefore, this study aimed to evaluate the survival and causes of death in patients with PFFs >5 years after surgery.

## 2. Patients and methods

### 2.1. Patients

This retrospective study included 123 patients (18 males and 105 females) with PFFs who underwent operations in our department between January 2014 and December 2016. The median age of the patients was 90 (range, 65–106) years. In total, 38 femoral neck fractures (FNFs) and 85 intertrochanteric fractures (IFs) were treated surgically, with 35 bipolar head arthroplasties and 3 screw fixations performed for FNFs and 85 proximal femoral nail antirotations (PFNA) performed for IFs. The mean follow-up time after surgery was 58.9 (range: 1–106) months. This study was conducted in compliance with the principles of the Declaration of Helsinki. The patients provided written consent, where possible. For patients who could not provide written consent, comprehensive consent was obtained. The current study was approved by Kushimoto Munincipal Hospital (approved number: 10001; approved date: December 12, 2022).

### 2.2. Methods of assessment

Overall survival (OS) and 1- and 5-year survival rates were evaluated. In addition, survival rates were compared between males and females, patients aged ≥90 and <90 years, and patients with IFs and FNFs. The presence of comorbidities was also assessed. Moreover, the waiting time after the injury (died vs survived), operative time (PFNA vs FNF; died vs survived), and blood loss (PFNA vs FNF; died vs survived) were calculated. In fatal cases at the final observation, the cause of death was investigated and compared between patients with IFs and FNFs, as well as at <1 and >1 year.

### 2.3. Statistical analysis

The log-rank test was used for statistical analysis. Statistical significance was set at *P* < .05. Analyses were performed using Stat Mate 5.05 (ATMS, Tokyo, Japan).^[10,11]^

## 3. Results

Table 1 shows the comorbidities of patients. Among all patients, 83.7% had comorbidities (IF, 90.5%; FNF, 81.5%). Among the patients who died and survived, 89.1% (IFs, 86.8%; FNFs, 100%) and 80.5% (IFs, 85.1%; FNFs, 76.6%), respectively, had comorbidities. The most common comorbidities were cardiac, renal, brain, and pulmonary diseases with 22, 10, 8, and 4 cases, respectively. Hypertension was the most common comorbidity, followed by chronic kidney disease and arrhythmia; diabetes mellitus was the third most common. A total of 90.5% and 81.5% of patients with IFs and FNFs, respectively, had comorbidities. A total of 86.8% and 100% of IF and FNF deaths, respectively, were associated with comorbidities. Moreover, 85.1% and 76.6% of the IF and FNF survivors, respectively, had comorbidities.

**Table 1:** Frequency of comorbidities at admission for each hip fracture type.

	FNF (n(%))	IF (n(%))
Senility	0 (0)	10 (11.7)
Heart failure	3 (8.5)	4 (4.7)
Lung disease	2 (5.7)	12 (14.1)
Abdominal aortic aneurysm	2 (5.7)	2 (2.3)
Renal failure	0 (0)	2 (2.3)

FNF = femoral neck fracture, IF = intertrochanteric fracture.

**Figure 1.**
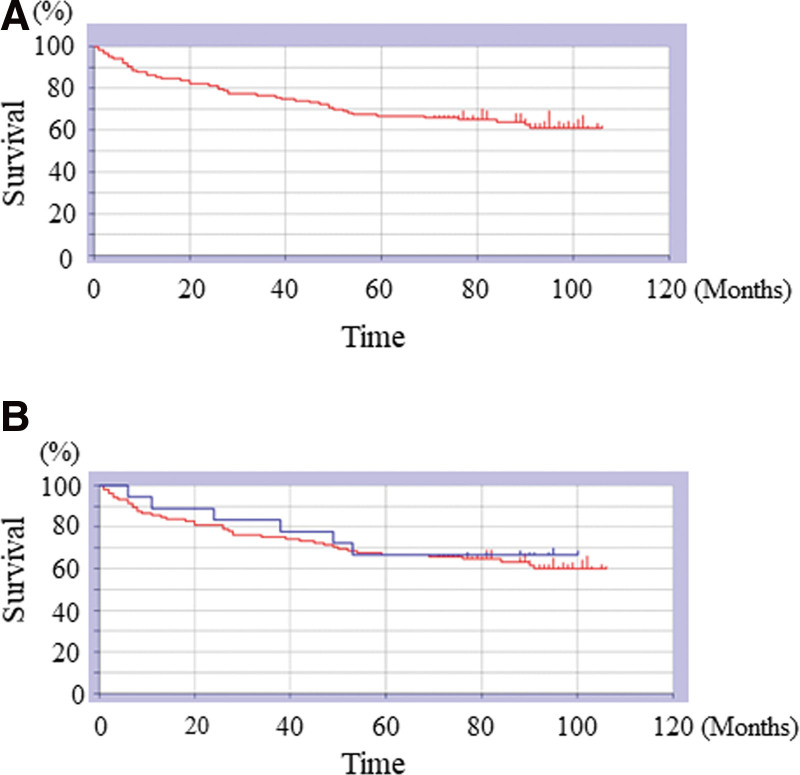
(A) Kaplan–Meier curves showing survival rates. The overall survival rates at 1 and 5 yr were 88.9% and 66.7%, respectively. (B) Kaplan–Meier curves showing survival rates. The 1- and 5-yr male/female OS rates were 88.8%/88.3% and 66.6%/66.6%, respectively (*P* = .89). OS = overall survival.

**Figure 2.**
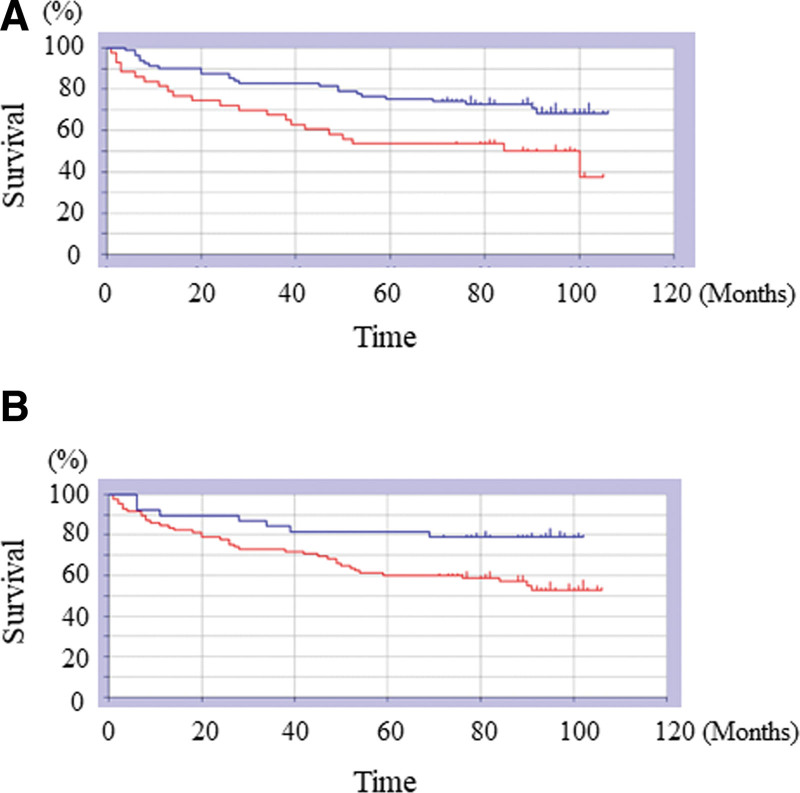
(A) Kaplan–Meier curves showing survival rates. The 1- and 5-yr OS rates for the <90/≥90 yr age groups were 90.1%/76.7% and 75.3%/53.4%, respectively (*P* < .01). (B) Kaplan–Meier curves showing survival rates. The 1-yr OS was 85.7%88.8% (IF/FNF), and the 5-yr OS was 60%/81.5% (IF/FNF). Patients with IFs had significantly lower OS than those with FNFs at both 1 and 5 yr (*P* = .015). FNFs = femoral neck fractures, IF = intertrochanteric fractures, OS = overall survival.

The 1- and 5-year survival rates of all patients were 88.9% and 66.7%, respectively (Fig. 1A). The mean survival time was 77.1 months. The survival rate at the last follow-up was 37.3% (46/123). The 1-year survival rates in male and female patients were 88.8% and 83.8%, respectively, with no significant difference (*P* = .88). Five-year survival rates were 66.6% (not significant; *P* = .88; Fig. 1B) for both male and female patients. Mean survival was 76.7 and 76.4 months for male and female patients (n = 105), respectively. The 1- and 5- year survival rates were 90.1/76.7% and 53.4/75.3%, respectively, for the ≥90/< 90 years age groups (Fig. 2A). Patients aged ≥90 years had a significantly poorer prognosis than those aged <90 years (*P* < .01). Mean survival was 64.9 and 84.6 months for the patients aged ≥90 and <90 years, respectively. The 1-year survival rates for patients with IFs and FNFs were 85.7% and 88.8%, respectively (Fig. 2B). The 5-year survival rates for patients with IFs and FNFs were 60% and 81.5%, respectively. Patients with IFs had a significantly poorer prognosis than those with FNFs (*P* = .015). The mean survivals were 71.8 and 85.7 months among patients with IFs (n = 85) and FNFs (n = 38), respectively. Table 2 shows the causes of death among patients with IFs and FNFs. The overall waiting time after the injury was 2 ± 1.5 (days; mean ± S.D.), that of died cases was 2 ± 1.59 (days; mean ± S.D.), and that of survived cases was 2 ± 1.54 (days; mean ± S.D.). There was no significant difference of waiting time after the injury between died and survived cases (*P* = 1.0). The overall operated time was 53 ± 24.9 (minute; mean ± S.D.), that of PFNA cases was 44 ± 22.8 (minute; mean ± S.D.), and that of FNF cases was 75 ± 18.2 (minute; mean ± S.D.). The operative time of FNF cases was significantly longer than that of PFNA cases (*P* < .001). The overall blood loss was 10 ± 76.0 (mL; mean ± S.D.), that of PFNA cases was 10 ± 33.1 (mL; mean ± S.D.), and that of FNF cases was 97.5 ± 112.7 (mL; mean ± S.D.). The blood loss of FNF cases was significantly larger than that of PFNA cases (*P* < .001). The operative time of died cases was 43.5 ± 24 (minute; mean ± S.D.), whereas that of survived cases was 60 ± 24.4 (minute; mean ± S.D.). The operative time of survived cases was significantly longer than that of died cases (*P* < .001). The blood loss of died cases was 10 ± 114.8 (mL; mean ± S.D.), whereas that of survived cases was 10 ± 41.7 (mL; mean ± S.D.). There was no significant difference of blood loss between died and survived cases (*P* = 1.0). The overall causes of death were natural causes (10 cases), aspiration pneumonia (9 cases), bronchopneumonia (6 cases), worsening heart failure (5 cases), acute myocardial infarction (4 cases), abdominal aortic aneurysm (4 cases), lung cancer 2 cases), and chronic renal failure (2 cases). The leading causes of death among patients with IFs were natural causes (10 cases) and aspiration pneumonia (7 cases; Table 2). Among patients with FNFs, the leading causes of death were abdominal aortic aneurysm (2 cases) and acute myocardial infarction (2 cases; Table 2). The leading causes of death within 1 year included aspiration pneumonia (5 cases) and natural causes (3 cases; Table 3). The leading causes of death ≥1 year included senility (7 cases), bronchial pneumonia (6 cases), and aspiration pneumonia (4 cases; Table 3).

**Table 2:** Cause of death among patients with IF and FNF.

	IF (n (%))	FNF (n (%))
Senility	10 (31.2)	0 (0)
Aspiration pneumonia	6 (18.7)	1 (12.5)
Bronchial pneumonia	6 (18.7)	0 (0)
Heart failure exacerbation	4 (12.5)	1 (12.5)
Acute myocardial infarction	2 (6.25)	2 (25.0)
Abdominal aortic aneurysm	2 (6.25)	2 (25.0)
Chronic kidney failure	2 (6.25)	0 (0)
Cerebral hemorrhage	0 (0)	1 (12.5)
Lung cancer	0 (0)	1 (12.5)

FNF = femoral neck fracture, IF = intertrochanteric fracture.

**Table 3:** Causes of death <1 yr and ≥1 yr after surgery.

	<1 yr (n (%))	≥1 yr (n (%))
Aspiration pneumonia	5 (29.4)	4 (14.8)
Senility	3 (17.6)	7 (25.9)
Heart failure exacerbation	2 (11.7)	3 (11.1)
Abdominal aortic aneurysm	2 (11.7)	2 (7.4)
Pulmonary embolism	1 (5.8)	0 (0)
Intestinal obstruction	1 (5.8)	0 (0)
Acute myocardial infarction	1 (5.8)	3 (11.1)
Lung cancer	1 (5.8)	0 (0)
Colon cancer	1 (5.8)	0 (0)
Bronchial pneumonia	0 (0)	6 (22.2)
Chronic kidney failure	0 (0)	2 (7.4)

## 4. Discussion

In 2007, Japan had approximately 150,000 PFF cases.^[12]^ Epidemiological studies have shown that the incidence of PFFs increases gradually with age, beginning at the age of 40 years and increasing rapidly after 75 years of age.^[12]^ The most common comorbidities reported in patients with PFFs are hypertension, diabetes mellitus, and heart failure.^[4,5]^ In addition to these, renal disease was common in this study. The average age of patients with PFF is approximately 80 years, and more than 75% of femoral fractures occur in patients aged >75 years. The present study reports the long-term results of PFFs in older patients (average age, 90 years) and a survey of causes of death. The mortality rate among patients with PFFs at 1-year postoperatively is approximately 15% to 25%, as previously described.^[13–15]^ The mortality rate was 27% when restricted to those aged ≥ 80 years.^[5]^ In addition, previous reports have shown a mortality rate of approximately 30% to 50% at 5 years postoperatively.^[5]^ The 5-year mortality rate of patients aged >80 years has also been reported to be approximately 30%.^[5]^ The results of the current study were generally favorable, despite the average age of the patients being 90 years. According to previous reports, during the follow-up period, the age-adjusted mortality rate after surgery was higher in men than in women.^[12–15]^ Regardless of age, higher mortality rates 1 year after injury have been reported among male patients than among female patients.^[16]^ Mortality rates have been strongly correlated with older age (>90 years).^[17]^ Mortality rates have also been reported to be highest among patients aged >85 years.^[4]^ However, patients aged >90 years have been reported to have better survival rates at 18 months after surgical treatment.^[4]^ In this study, the mean age of the patients was 90 years. The survival rate was also extensively lower in those aged ≥90 years than in those aged <90 years. The most common cause of death was senility, followed by pneumonia. These findings suggest that the life expectancy may have influenced the results of this study and that age may not be a prognostic factor. Mortality rates are reported to be higher for IFs at 1-year postoperatively than for FNFs.^[8]^ In addition, although IFs and FNFs had similar mortality rates at 1 year, IFs had considerably higher rates at 5 and 10 years.^[5,8]^ The poorer prognosis has been attributed to the fact that patients with IFs were usually older, frailer, less functionally capable, and had more comorbidities than those with FNFs.^[18]^ The difference in mortality rates between IFs and FNFs has also been attributed to intrinsic changes in pre-fracture comorbidities and functional status.^[19]^ The mortality in the first year after fracture has been associated with surgical complications and trauma.^[20]^ In contrast, the long-term mortality has been attributed to comorbidities and worsening health status due to activity limitations and disuse.^[21]^ The most common comorbidities reported after PFFs are hypertension, diabetes, and heart failure.^[4,5]^ Many patients in this study also had comorbidities, which were directly related to the cause of death. These results suggest that perioperative control of comorbidities is important for patients with preoperative comorbidities. There is no safe limit to the waiting time before surgery for hip fracture patients.^[22]^ It has been reported that the risk increases with each hour of waiting time for surgery.^[22]^ Moreover, Surgical time for FNA is reported to be longer than for PFNA, and blood loss is reported to be higher. It has also been reported that early mortality may increase due to increased blood loss and operative time.^[23]^ In this study, only the operative time and blood loss for PFNA and FNF supported previous reports. Further studies with a larger number of patients are warranted. In general, the most common causes of death among persons aged ≥65 years are heart disease, malignancy, lung disease, stroke, myocardial infarction, and dementia.^[24]^ The most common causes of death in patients with PFFs are cardiac disease, malignant neoplasms, trauma, respiratory disease, and metabolic disease.^[8]^ Other reports indicate that infection, pulmonary embolism, and heart failure are the major fatal complications, and in patients undergoing PFF surgery, the most common cause of death is cardiovascular disease, followed by dementia and Alzheimer disease.^[15]^ Men were also more likely to die from respiratory diseases, malignant neoplasms, and cardiovascular disease than women.^[15]^ Although mortality in the first year after fracture is associated with complications from surgery and trauma, long-term mortality has been attributed to a worsening health status owing to comorbidities and disuse.^[20,21]^ The cause of death that was relatively well characterized in the current study was pneumonia. Aspiration pneumonia and bronchopneumonia were the most common short- and long-term causes of death, respectively. Furthermore, pneumonia and abdominal aortic aneurysm are common causes of death after IF surgery. Abdominal aortic aneurysm is a common cause of death after FNF surgery. These findings indicate that pneumonia requires careful follow-up during both the early and late postoperative periods. The management of hypertension and abdominal aortic aneurysms may be important for achieving favorable outcomes.

### 4.1. Limitations

The current study had few limitations. These included a small number of male patients, cervical fracture cases, lack of a postoperative functional assessment, and lack of coordination regarding the implant selection, fracture type, and rehabilitation. Furthermore, Kushimoto Town is located at the southernmost tip of Honshu, and its regional specificity makes it difficult to generalize.^[25]^ However, the outcome of certain PFFs could be studied with respect to the survival and cause of death using a statistically unproblematic number of cases. However, we believe that the strength of this study relies within its a long-term observational design with a focus on the long-term survival and cause of death.

### 4.2. Conclusion

This study reports the long-term postoperative results of patients who underwent PFF surgery. These results suggest that controlling hypertension-related comorbidities and screening and managing pneumonia may improve the long-term prognosis, even in the long-term postoperative period.

## Author contributions

**Conceptualization:** Kazuhiko Hashimoto, Yu Shinyashiki, Ryosuke Kakinoki, Masao Akagi.

**Data curation:** Kazuhiko Hashimoto, Yu Shinyashiki, Ryosuke Kakinoki.

**Formal analysis:** Kazuhiro Ohtani, Ryosuke Kakinoki.

**Investigation:** Kazuhiko Hashimoto, Yu Shinyashiki, Kazuhiro Ohtani.

**Methodology:** Kazuhiko Hashimoto.

**Project administration:** Kazuhiko Hashimoto, Ryosuke Kakinoki.

**Resources:** Kazuhiko Hashimoto, Yu Shinyashiki.

**Software:** Kazuhiko Hashimoto.

**Supervision:** Kazuhiko Hashimoto, Kazuhiro Ohtani, Ryosuke Kakinoki, Masao Akagi.

**Validation:** Kazuhiko Hashimoto, Yu Shinyashiki, Kazuhiro Ohtani, Ryosuke Kakinoki, Masao Akagi.

**Visualization:** Kazuhiko Hashimoto, Kazuhiro Ohtani, Masao Akagi.

**Writing – original draft:** Kazuhiko Hashimoto, Yu Shinyashiki, Kazuhiro Ohtani, Ryosuke Kakinoki, Masao Akagi.

**Writing – review & editing:** Kazuhiko Hashimoto, Yu Shinyashiki, Kazuhiro Ohtani, Ryosuke Kakinoki, Masao Akagi.
